# Reducing Oxygen Stress and Improving Hydrogen Availability Boosts Microbial Electrosynthesis by *Clostridium* *ljungdahlii*

**DOI:** 10.1002/cssc.202501118

**Published:** 2025-09-11

**Authors:** Anne Kuchenbuch, Sara Al‐Sbei, Luis F. M. Rosa, Santiago T. Boto, Martin Westermann, Miriam A. Rosenbaum, Falk Harnisch

**Affiliations:** ^1^ Department of Microbial Biotechnology Helmholtz‐Centre for Environmental Research GmbH–UFZ Permoserstr. 15 04318 Leipzig Germany; ^2^ Department Bio Pilot Plant Leibniz Institute for Natural Product Research and Infection Biology–Hans‐Knöll‐Institute Beutenbergstr. 11a 07745 Jena Germany; ^3^ Faculty of Biological Science Friedrich Schiller University Jena Bachstr. 18k 07743 Jena Germany; ^4^ Present address: Biological and Environmental Science and Engineering Division (BESE) King Abdullah University for Science and Technology (KAUST) Thuwal 23955‐6900 Saudi Arabia; ^5^ Electron Microscopy Center Jena University Hospital Ziegelmühlenweg 1 07743 Jena Germany

**Keywords:** bioprocess engineering, *Clostridium ljungdahlii*, electrobioreactors, green chemistry, microbial electrosynthesis

## Abstract

Microbial electrosynthesis (MES) holds great promise for converting carbon dioxide (CO_2_) into building blocks of the (bio)chemical industry. Its advancement is hindered by limited process control and an incomplete understanding of the oxygen (O_2_) stress response of biocatalysts or key engineering parameters like the availability of hydrogen (H_2_). With *Clostridium ljungdahlii* as a model acetogen for strict anaerobic MES from CO_2_, the effect of O_2_ stress and H_2_ availability using 1‐L electrobioreactors is showcased, providing high process control and relevance for follow‐up engineering and scaling. Using a combinatorial approach of two cathode materials, three anode types, and various current regimes ranging from −5 to −80 mA, MES performance is boosted by overcoming O_2_ stress and insufficient H_2_ distribution at high current. It is demonstrated that a large‐surface‐area carbon fiber fabric cathode combined with O_2_ evolution anodes flushed with nitrogen (N_2_) allows the highest reproducible acetate concentration of 12.44 ± 1.56 g L^−1^ and maximum acetate production rate of 0.6 ± 0.1 g L^−1^ d^−1^ reported for MES from CO_2_ using a pure culture. There is certainly room for improved process control at this and even larger scales, showing that the ceiling of strict anaerobic MES is far from being reached.

## Graphical abstract

Microbial electrosynthesis (MES) from CO_2_ by acetogens enables the production of value‐added chemicals. However, its current limitations include O_2_ stress and insufficient H_2_ availability. Using *Clostridium ljungdahlii* as a model in 1‐L electrobioreactors providing high process control, MES is boosted to unprecedent acetate concentrations and rates through a combinatorial approach involving different electrode materials and current regimes.

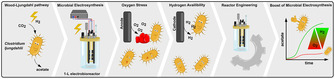

## 1 Introduction

The current economy that is based on linear use of fossil resources needs to be transitioned into a circular economy built on renewable sources.^[^ ^1^ ^]^ This transition of the material base of our welfare will lead to societal sustainability and increased resilience. Specifically, the material base for carbon feedstock needs to shift from fossil oil and gas toward renewable feedstocks, as well as carbon dioxide (CO_2_). Beyond abandoning the extraction of non‐renewable resources and tapping into natural resources, alternative use cycles that lead to increased carbon residence times in the technosphere are required. In conjunction with the material base, the foundation of the energy input needs to transition toward renewable sources, e.g., photovoltaics or wind power.^[^ ^2^ ^]^ The transition of both realms, the material and energy base, can be addressed when replacing petrochemical refineries with electrobiorefineries. When it comes to the production of chemicals, one special key element under the umbrella of the electrobiorefinery concept is microbial electrosynthesis (MES).^[^ ^3^ ^]^

MES couples the use of renewable electric power with microbial production.^[^ ^4^ ^]^ Most prominently, MES addresses producing value‐added compounds from CO_2_ as a carbon source. MES from CO_2_ is based mainly on anaerobic, autotrophic microorganisms—prominently acetogens—that take up electrons from the cathode for CO_2_ fixation by the Wood–Ljungdahl pathway.^[^ ^5^ ^]^ Electrons are introduced into metabolism directly through specialized molecular machinery present in some bacteria, or with the mediation of electron shuttles like hydrogen (H_2_). Despite the huge interest in MES research, the performance of MES by acetogenic microorganisms is considered low in terms of growth rates and productivity when compared to established gas fermentation.^[^ ^6^ ^]^ MES is usually performed in two‐chamber lab‐scale reactors, e.g., H‐cell type reactors, where a cation exchange membrane separates the anode and the cathode compartment. The membrane is essential to prevent the oxygen (O_2_) evolving at the anode by water splitting from reaching the anaerobic biocatalyst at the cathode.^[^ ^7^ ^]^ However, the membrane and non‐standardized reactor architecture still provide plenty of entry points for O_2_. Thus, the cathodic chamber cannot be considered to be completely shielded from O_2_, which can be one reason contributing to the low performance of MES from CO_2_.^[^ ^8^ ^]^ Also, the mode of electron transfer during MES has been highly debated in the past decade and certainly needs specific clarification for each biocatalyst. A recent work for the commonly used pure‐culture biocatalyst *Clostridium ljungdahlii* strongly indicates an essential H_2_‐mediated process.^[^ ^9^ ^]^ Thus, H_2_ limitation needs to be considered as a main reason for low MES performance with this bacterium and should be counteracted with electrobioprocess design.

Besides the low performance, the low economic value of the product portfolio being limited to methane, acetate, and ethanol has been a major obstacle for the advancement of MES.^[^ ^10^ ^]^ We previously reported that higher value‐added products, such as glycine and ethanolamine, can be obtained using MES from CO_2_ with *C. ljungdahlii* pure cultures.^[^ ^9^ ^]^ Most often, undefined mixed cultures are the preferred choice in MES due to their simplicity, resilience, and high performance. However, pure cultures generally exhibit a more consistent acetate production, reproducibility, as well as improved process control, and the opportunity to further expand the product portfolio via, for example, gene editing.^[^ ^11^ ^]^

Continuous stirred tank reactors (CSTRs) are workhorses of the biotechnological industry, and the liter‐scale is often the lowest volume considered relevant for process engineering. We have established an electrobioreactor platform that is based on adapting commercial CSTR‐based bioreactors with an upgrade kit that enables electrochemical process control and monitoring.^[^ ^12^, ^13^, ^14^ ^]^ In this study, we extended the operation of these new electrobioreactors for cathodic control of microbial reactions. Using 1‐L electrobioreactors, we investigated limitations for MES from CO_2_ using *C.* *ljungdahlii*, focusing on strategies 1) to avoid O_2_ crossover from the anode to the cathode and 2) to overcome H_2_ limitation in the cathode chamber. Therefore, we explored different technical solutions to avoid the O_2_ crossover through the cation exchange membrane from the anode to the cathode compartment. Strategies such as flushing N_2_ gas into the anode compartment to oust the produced O_2_ and implementing activated carbon felt electrodes as sacrificial anodes to completely avoid O_2_ production as proposed by Rohbohm et al. (2023) were evaluated.^[^ ^14^ ^]^ To tackle the issue of limited H_2_ availability in the cathode chamber, we substituted carbon rod electrodes with customized woven carbon fiber electrodes, providing a larger active surface area while also increasing the spatial distribution of H_2_ in the electrobioreactors by a novel 3D‐architecture.^[^ ^15^ ^]^ Compared to monolithic rods, carbon fiber fabric electrodes achieve lower current density (at equivalent total current) through a higher electrode‐area‐to‐reactor‐volume ratio and thus enable a better distribution of the equivalent H_2_ amount and hence an improved availability for *C. ljungdahlii*.

## 2 Results and Discussion

### 2.1 Oxygen Crossover to the Cathode Decreases MES Performance and Leads to Oxidative Stress

**Figure 1:**
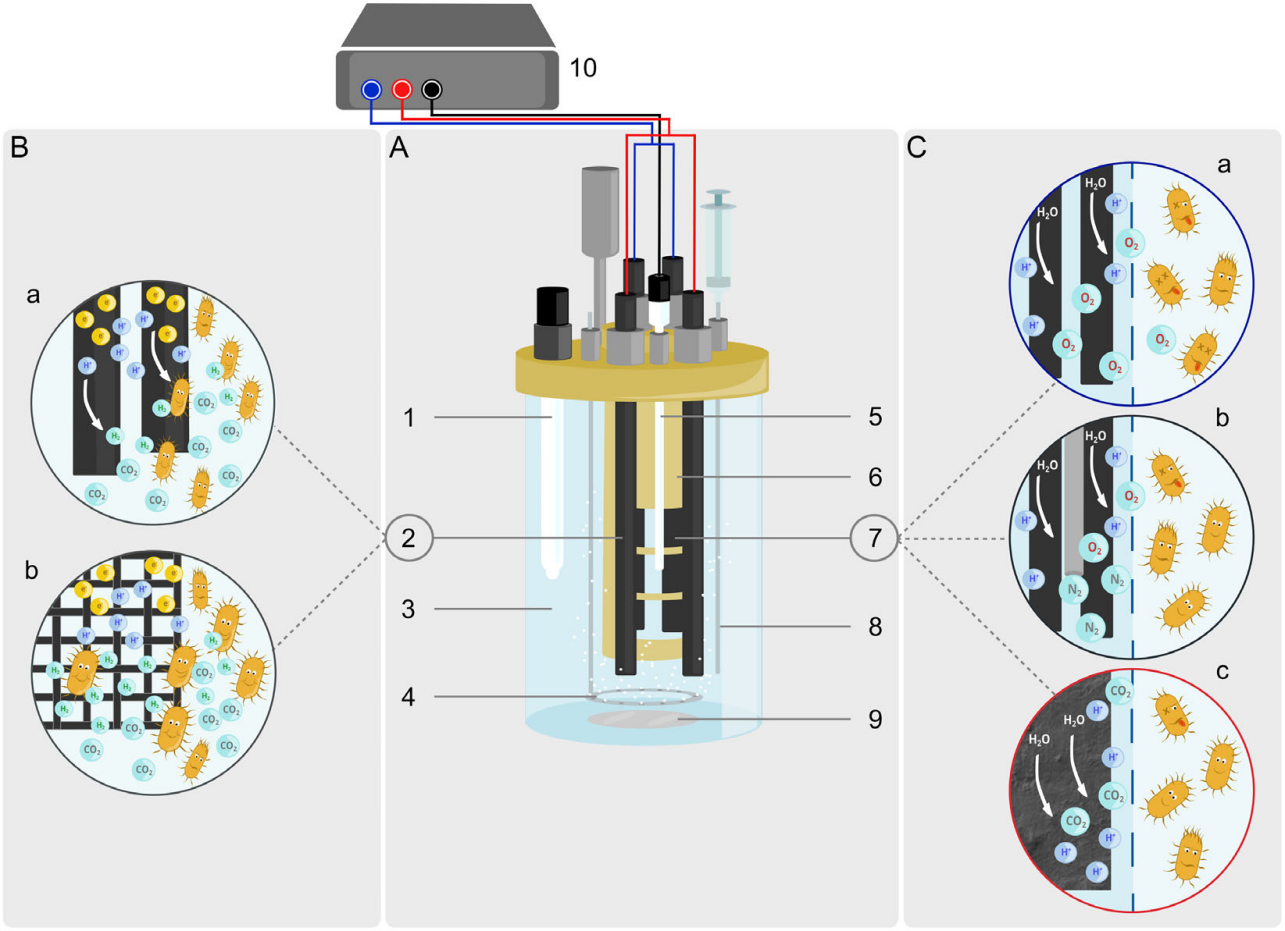
A) A schematic illustration of a 1‐L electrobioreactor with 1) pH sensor, 2) WE (i.e., cathode), 3) WE chamber (i.e., cathode chamber), 4) gas sparger, 5) reference electrode, 6) CE chamber (i.e., anode chamber) with membrane windows, 7) CE (i.e., anode), 8) sampling port, and 9) stirrer under 10) galvanostatic control; B) used cathode materials for H_2_ evolution reaction: (a) carbon rod and (b) carbon fiber fabric material; and C) used anodes: (a) OER anode, (b) OER anode flushed with N_2_, and (c) sacrificial anode. The combination of the two cathode materials under B and the three anodes under C enables to investigate six different electrobioreactor configurations (see Table 1).

MES from CO_2_ using *C. ljungdahlii* requires strictly anaerobic conditions at the cathode. However, O_2_ is produced by the electrolytic splitting of water via the oxygen evolution reaction (OER, 2H_2_O → O_2_ + 4H^+^ + 4e^−^) at the anode. This O_2_ passes the membrane into the cathode chamber, as we also confirmed for two exemplary cation exchange membranes (see SI 1.1). Any extended exposure of *C. ljungdahlii* to O_2_ leads to oxidative stress, either by O_2_ itself or by the formation of superoxide and hydrogen peroxide.^[^ ^16^ ^]^ In addition, abiotic and biotic cathodic reactions with O_2_ can impair MES performance and cause a decrease in Coulombic efficiency (CE), e.g., via the oxygen reduction reaction (O_2_ + 4H^+^ + 4e^−^ → 2H_2_O) and further stress by hydrogen peroxide formation (O_2_ + 2H^+^ + 2e^−^ → 2H_2_O_2_). As a consequence, even small traces of O_2_ in the cathode chamber may reduce the performance of MES using strict anaerobes.^[^ ^8^ ^]^ To investigate the influence of O_2_ crossover on MES, six different setups of electrobioreactors (**Figure** **1**A) consisting of different electrode materials for cathode and anode (Figure 1B,C, **Table** **1**) were compared in terms of microbial growth (i.e., OD_600 nm_) and the metabolic performance (i.e., production of acetate in g L^−1^; glycine and ethanolamine in mg L^−1^). Galvanostatic operation with a fixed current of initially −5 mA, and subsequently −20 mA, was applied with N_2_ and CO_2_ being continuously flushed to the cathode chamber.

**Table 1:** An overview of the different electrobioreactor setups, highlighting the respective cathode and anode materials used in each electrobioreactor configuration.

Setup	Cathode material	Anode material
Carbon rod cathode, OER anode	Carbon rod	Carbon rod
Carbon rod cathode, OER anode, N_2_ flushed	Carbon rod	Carbon rod, flushed with N_2_
Carbon rod cathode, Sacrificial anode	Carbon rod	Activated carbon felt
Carbon fiber cathode, OER anode	Carbon fiber fabric material	Carbon rod
Carbon fiber cathode, OER anode, N_2_ flushed	Carbon fiber fabric material	Carbon rod, flushed with N_2_
Carbon fiber cathode, Sacrificial anode	Carbon fiber fabric material	Activated carbon felt

**Figure 2:**
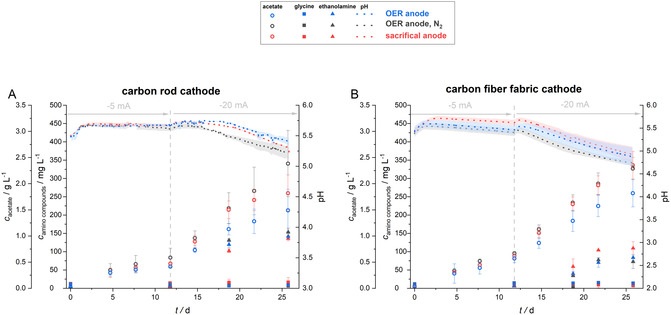
MES from CO_2_ using *C. ljungdahlii*: The figure illustrates the concentration of acetate and of the amino compounds glycine and ethanolamine as well as the development of pH in electrobioreactors under galvanostatic operation. The electrobioreactors were operated for 12 days at a current of −5 mA and further 14 days at −20 mA. Two types of cathodes were used: A) carbon rod cathodes and B) carbon fiber fabric cathode. Each cathode type was combined with three different anodes: an OER anode (blue), an OER anode flushed with N_2_ (black), and a sacrificial anode (red) The error bars and shaded areas represent the standard deviation from mean values (*n* = 3).

After 12 days of galvanostatic operation with −5 mA, no clear difference in performance with the two different cathode materials was noticed for all three anodes (**Figure** **2**). Acetate concentrations of the different electrobioreactor setups were between 0.42 and 0.67 g L^−1^. After increasing the current to −20 mA followed by 14 days more of operation, a clear variation in the performance was observed (**Table** **2**). When considering OER anodes, an acetate production of only 1.49 ± 0.41 g L^−1^ for carbon rod cathodes and 1.82 ± 0.26 g L^−1^ for carbon fiber fabric cathodes was detected. When using OER anodes flushed with N_2_ or a sacrificial anode, where CO_2_ instead of O_2_ is produced, a higher total acetate production was achieved in MES, both using carbon rod cathodes and carbon fiber fabric cathodes. Final acetate concentrations of 2.38 ± 0.63 g L^−1^ (carbon rod cathode/OER anode, N_2_ flushed) and 1.82 ± 0.35 g L^−1^ (carbon rod cathode/sacrificial anode), as well as 2.29 ± 0.21 g L^−1^ (carbon fiber fabric cathodes/OER anode, N_2_ flushed) and 2.35 ± 0.26 g L^−1^ (carbon fiber fabric cathode/sacrificial anode) were reached, respectively. This shows that the negative influence of the O_2_ passing the cation exchange membrane into the cathode chamber decreased the acetate production. Maximal OD_600 nm_ of 0.147 ± 0.011 (OER anode), 0.137 ± 0.040 (OER anode, N_2_ flushed), and 0.179 ± 0.038 (sacrificial anode) for carbon rod cathode electrobioreactors (Figure S2, Supporting Information) as well as of 0.112 ± 0.049 (OER anode), 0.115 ± 0.019 (OER anode, N_2_ flushed), and 0.122 ± 0.053 (sacrificial anode) for carbon fiber fabric cathode electrobioreactors (Figure S3, Supporting Information) were reached. The consistently lower OD in the fabric electrode systems might indicate a possible accumulation of *C. ljundahlii* biomass on the more spatially distributed carbon fabric network.

**Table 2:** The concentration of acetate as the main product of the MES from CO_2_ using *C. ljungdahlii* as well as the amino compounds glycine and ethanolamine. The concentrations are presented the different electrobioreactor setups under galvanostatic operation. The electrobioreactors were operated for 12 days at a current of −5 mA and further 14 days at −20 mA. Two types of cathodes were used, carbon rod cathodes and carbon fiber fabric cathode. Each cathode type was combined with three different anodes: an OER anode, an OER anode flushed with N_2_ and a sacrificial anode. The values shown represent the mean ± standard deviation (*n* = 3).

Cathode	Anode	Acetate [g L^−1^]		Glycine [mg L^−1^]		Ethanolamine [mg L^−1^]	
		−5 mA, 12 d	−20 mA, 14 d	−5 mA, 12 d	−20 mA, 14 d	−5 mA, 12 d	−20 mA, 14 d
Carbon rod	OER	0.42 ± 0.03	1.49 ± 0.41	11.3 ± 0.2	8.7 ± 4.4	6.2 ± 0.5	141.7 ± 7.8
	OER, N_2_	0.59 ± 0.18	2.38 ± 0.63	12.5 ± 0.8	7.3 ± 5.2	7.3 ± 0.1	153.5 ± 10.6
	Sacrificial	0.47 ± 0.03	1.82 ± 0.35	14.3 ± 1.3	16.3 ± 13.0	3.9 ± 1.2	136.1 ± 6.3
Carbon fiber fabric	OER	0.57 ± 0.08	1.82 ± 0.26	14.6 ± 1.6	13.8 ± 1.1	6.1 ± 2.1	83.7 ± 7.6
	OER, N_2_	0.67 ± 0.04	2.29 ± 0.21	12.9 ± 4.1	8.1 ± 5.1	7.2 ± 3.8	72.5 ± 18.8
	Sacrificial	0.62 ± 0.06	2.35 ± 0.26	14.4 ± 2.3	8.9 ± 5.1	5.9 ± 2.7	109.4 ± 17.9

Besides acetate as the main product of MES from CO_2_ with *C. ljungdahlii*, Boto et al. (2023)^[^ ^9^ ^]^ described the two amino compounds, glycine and ethanolamine being central metabolites, as products with so far unprecedented high titers in MES of 390 mg L^−1^ glycine and 140 mg L^−1^ ethanolamine. During our experiment, glycine with an initial concentration of 11.8 ± 0.8 mg L^−1^, likely originating from the provided yeast extract in the media, did not accumulate in the experiment (between 11.3 ± 0.2 and 14.6 ± 1.6 mg L^−1^ after 12 days of operation at −5 mA; between 7.3 ± 5.2 and 16.3 ± 13.0 mg L^−1^ after further 14 days of operation at −20 mA). In contrast, the formation of ethanolamine in all electrobioreactor configurations was confirmed. The initial concentration of 0.17 ± 0.26 mg L^−1^ increased slightly to between 3.9 ± 1.2 and 7.3 ± 0.1 mg L^−1^ after 12 days at −5 mA for the different electrobioreactor setups. After the switch to −20 mA, a clear boost in ethanolamine production was observed. Thereby, the increase with carbon rod cathodes was higher with 141.7 ± 7.8 mg L^−1^ (OER anode), 153.5 ± 10.6 mg L^−1^ (OER anode, N_2_ flushed), and 136.1 ± 6.3 mg L^−1^ (sacrificial anode) compared with carbon fiber fabric cathodes with 83.7 ± 7.6 mg L^−1^ (OER anode), 72.5 ± 18.8 mg L^−1^ (OER anode, N_2_ flushed), and 109.4 ± 17.9 mg L^−1^ (sacrificial anode). The production of glycine and ethanolamine has not been observed for metabolically related gas fermentation with *C. ljungdahlii* and is hypothesized to be linked to redox stress during MES. Boto et al. (2023)^[^ ^9^ ^]^ assumed that glycine and ethanolamine production results from a high NADPH/NADP^+^ ratio imbalance caused by metabolic flux disruptions under stressed conditions. This was demonstrated using an in silico model but has not been experimentally verified yet.

The experiment clearly confirmed that the MES process and culture performance of *C. ljundahlii* were limited by the energy input. The increase of the applied current from −5 to −20 mA provided more H_2_ into the system, which increased the biocatalytic acetate production. Surprisingly, for the current regime of −5 mA for 12 days and further −20 mA for 14 days, it was not possible to determine a clear influence of the different cathode materials on the MES performance. Final acetate concentrations of 1.49 ± 0.41 g L^−1^ for carbon rod cathodes and 1.82 ± 0.35 g L^−1^ for carbon fiber fabric cathodes combined with OER anodes, as well as 2.38 ± 0.63 and 2.29 ± 0.21 g L^−1^, respectively, combined with OER anodes flushed with N_2_ showed no difference. Nevertheless, a comparison of the final acetate concentration with the carbon rod cathodes and the carbon fiber fabric cathodes, both combined with the sacrificial anodes, 1.82 ± 0.35 and 2.35 ± 0.26 g L^−1^, gave first indications that spatial H_2_ distribution could influence the performance of cathodic MES from CO_2_.

The CE (**Table** **3**) that can be considered as an overall performance parameter was only 54.8 ± 9.6% for the electrobioreactors equipped with carbon rod cathodes when OER anodes were used, but considerably higher for anodes not leading to O_2_ intrusion into the cathode chamber with 86.0 ± 15.5% (carbon rod cathode/OER anode, N_2_ flushed). The same holds true for carbon fiber fabric cathodes combined with OER anodes, where only a CE of 66.1 ± 8.3% was achieved, which is considerably lower compared to CEs using other anodes, i.e., 82.0 ± 5.6% (carbon fiber fabric cathode/OER anode, N_2_ flushed) and 85.4 ± 7.3% (carbon rod cathode/sacrificial anode). These results further support that O_2_ crossover hampered MES performance, which is caused by either oxidative stress or electrochemical short circuiting of O_2_ reacting with the cathode directly, which can be avoided by flushing the anode chamber with N_2_ or using sacrificial anodes. Surprisingly, using sacrificial anodes with carbon rod cathodes yielded a CE of only 66.2 ± 8.1%. Thus, all further experiments were performed with OER anodes flushed with N_2_ due to the simpler preparation process, fewer sources of errors, and a higher mechanical stability of the rods compared to the sacrificial anodes. Besides the CE of the entire experimental period, we also calculated the CE of the phases with different currents. In all electrobioreactors, a higher CE for applying −5 mA was achieved. CEs even exceeded 100%, except for 88.2 ± 4.4% for the carbon rod cathode and OER anode. These high values are likely a result of the initial co‐utilization of H_2_/CO_2_ and the provided low amount of yeast extract as an organic carbon source for biomass formation. Years of lab experiments have proven the benefit of addition of yeast extract for the initial biomass growth during autotrophic growth.^[^ ^17^ ^]^ Thus, the CE for the first 3–5 days does not solely represent the bioelectrochemical performance of the system. When the current was increased to −20 mA, an average decline in CE of more than half (52.4 ± 10.3%) was determined for all electrobioreactor configurations, showing that the produced H_2_ was not utilized efficiently due to a lack of active biocatalyst and was outgassed from the electrobioreactor with the CO_2_/N_2_ sparging.

**Table 3:** The CE of the MES from CO_2_ using *C. ljungdahlii* for the different electrobioreactor setups under galvanostatic operation. The electrobioreactors were operated for 12 days at a current of −5 mA and further 14 days at −20 mA. Two types of cathodes were used, carbon rod cathodes and carbon fiber fabric cathode. Each cathode type was combined with three different anodes: an OER anode, an OER anode flushed with N_2_, and a sacrificial anode. The calculation of the CE includes the formation of acetate, glycine, and ethanolamine, and values shown represent the mean ± standard deviation (*n* = 3).

Cathode	Anode	CE [%]		
		Complete, 26 d	−5 mA, 12 d	−20 mA, 14 d
Carbon rod	OER	54.8 ± 9.6	88.2 ± 4.4	47.8 ± 12.5
	OER, N_2_	86.0 ± 15.5	125.4 ± 28.4	77.7 ± 12.8
	Sacrificial	66.2 ± 8.1	100.5 ± 4.6	58.9 ± 8.9
Carbon fiber fabric	OER	66.1 ± 8.3	118.5 ± 14.9	54.9 ± 8.8
	OER, N_2_	82.0 ± 5.6	137.6 ± 7.0	70.1 ± 7.6
	Sacrificial	85.4 ± 7.3	130.3 ± 12.2	76.8 ± 7.4

**Figure 3:**
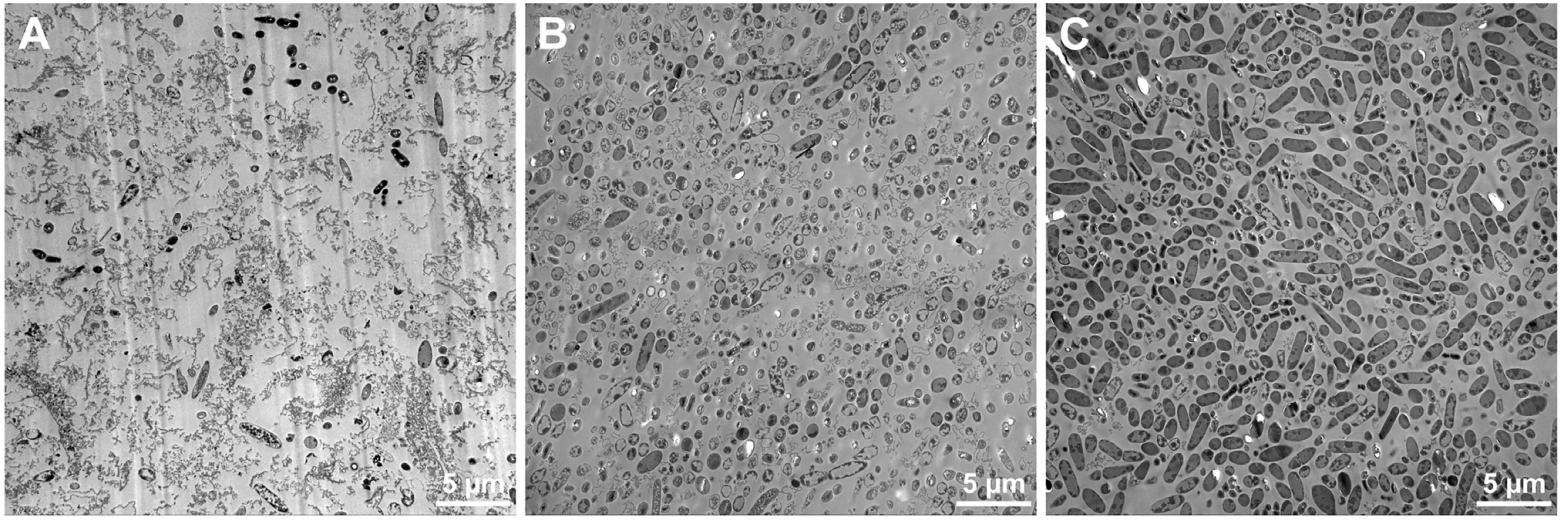
TEM images of *C. ljungdahlii* performing MES from CO_2_ in different electrobioreactor setups: A) electrobioreactor with carbon fiber fabric cathode material and OER anodes without O_2_ removal; B) electrobioreactor with carbon fiber fabric cathode material and OER anodes flushed with N_2_ for O_2_ removal; and C) electrobioreactor with carbon fiber fabric cathode material, O_2_ production in the anode chamber is avoided using the sacrificial anodes.

The effect of O_2_ crossover and thus oxidative stress was also observed by visual investigation of the microbial cells using transmission electron microscopy (TEM). Images were generated of *C. ljungdahlii* harvested from electrobioreactors equipped with carbon fiber fabric cathodes combined with either OER anodes (**Figure** **3**A), OER anodes flushed with N_2_ (Figure 3B), or with sacrificial anodes (Figure 3C), where different oxidative conditions were expected. When O_2_ was not removed or avoided, *C. ljungdahlii* showed clear indications of stress, with much lower intact cell counts, demonstrating how oxidative stress can significantly impact cell viability and highlighting the importance of preventing O_2_ from reaching the cathode chamber of electrobioreactors. This shows that decreasing O_2_ crossover to the cathode chamber, either by flushing the OER anode with N_2_ or the use of sacrificial anodes where OER is avoided, leads to decreased stress for *C. ljungdahlli* and therefore more viable cells.

### 2.2 3D Cathodes Allow Efficient MES at High Currents

The electrochemical H_2_ production in electrobioreactors takes place at the cathode surface. Hence, the geometry of the electrodes, the reactor design, and mass transfer impact the H_2_ distribution, residence time, and therefore availability to *C. ljungdahlii* for performing MES from CO_2_. As the mixing can only be improved to a certain limit using the standardized electrobioreactor infrastructure,^[^ ^18^ ^]^ the electrochemical production of H_2_ using a scalable 3D material is highly promising.^[^ ^15^ ^]^ In order to cover a large part of the electrobioreactor volume, a customized woven carbon fiber fabric cathode material forming a “reactive wall” was used (geometry: Figure S6, Supporting Information). This highly flexible yet stable cathode material was compared to carbon rod cathodes using the same absolute current that leads to different current densities and hence also local hydrogen concentrations. To assure comparability, both kinds of electrodes were combined with OER anodes flushed with N_2_, enabling anaerobic operation in the cathode chamber has already been proofed before. As shown in the Section 2.1, no difference in MES performance when using the two cathode materials and configurations was observed at a galvanostatic current of –5 or –20 mA. We hypothesized that at low current input, the small surface area of the carbon rod is still sufficient to allow an efficient transfer of H_2_ to the biocatalyst. However, at higher currents, an even distribution of the H_2_ evolution throughout the electrobioreactor with the carbon fiber fabric cathode should demonstrate its superior reaction characteristics in contrast to the localized H_2_ evolution with the carbon rod cathodes. Therefore, an experiment with increasing current steps, specifically –5, –20, –40, –60, and –80 mA for 7 days each, was performed.

**Figure 4:**
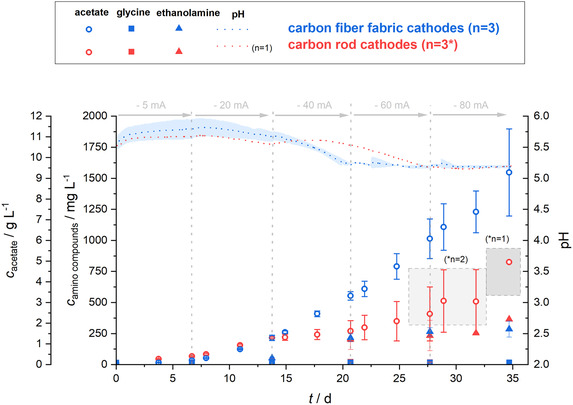
MES from CO_2_ using *C. ljungdahlii*: The figure illustrates the concentration of acetate and of the amino compounds glycine and ethanolamine as well as the development of pH in electrobioreactors under galvanostatic operation. The electrobioreactors were operated for 35 days with a stepwise current increase from −5 to −20, −40, −60, and −80 mA every 7 days. Two types of cathodes, carbon rod cathodes (red) and carbon fiber fabric cathode (blue), in combination with an OER anode flushed with N_2_ were used. The error bars and shaded areas represent the standard deviation of the mean (*n* = 3). Initially, each electrobioreactors setup was started in triplicates However, the carbon rod system failed for two replicates during the experiment (indicated by *n = *x* in the grey boxes).

Consistent with the previous results (Figure 2), the performance of *C. ljungdahlii* for MES from CO_2_ in terms of acetate production between carbon rod cathodes and carbon fiber fabric cathodes was again not different when operating at −5 and −20 mA. In fact, the acetate concentrations for the carbon rod cathodes of 1.29 ± 0.4 and 1.29 ± 0.12 g L^−1^ for carbon fiber fabric cathodes after 14 days (–5 mA for 7 d and −20 mA for 7 d) were similar (**Figure** **4**). In contrast, applying higher currents of −40, −60, and −80 mA led to an increased acetate concentration of 3.32 ± 0.21 g L^−1^ at −40 mA, 6.08 ± 0.96 g L^−1^ at −60 mA as well as 9.28 ± 2.10 g L^−1^ at −80 mA for the carbon fiber fabric cathodes, when compared to 1.61 ± 0.50 mg L^−1^ at −40 mA, 2.45 ± 1.31 g L^−1^ at −60 mA as well as 4.94 g L^−1^ (*n* = 1) at −80 mA for carbon rod cathodes (**Table** **4**). The final maximum concentration of 9.28 ± 2.10 g L^−1^ with this electrode architecture providing a surface‐area‐to‐reactor‐volume ratio of 166.7 cm^2^ L^−1^ was already reached after 35 days of operation. This is a great improvement compared to the benchmark Boto et al. (2023),^[^ ^9^ ^]^ who reported 6.06 g L^−1^ at an even larger surface‐area‐to‐reactor‐volume ratio of 382.63 cm^2^ L^−1^ after 55 days of potentiostatic operation at −0.9 V vs Ag/AgCl sat. KCl.

**Table 4:** The concentration of acetate as the main product of the MES from CO_2_ using *C. ljungdahlii* as well as the amino compounds glycine and ethanolamine. The concentrations are presented the different electrobioreactor setups under galvanostatic operation. The electrobioreactors were operated for 35 days with a stepwise current increase from −5 to −20, −40, −60, and −80 mA every 7 days. Two types of cathodes, carbon rod cathodes and carbon fiber fabric cathode, in combination with an OER anode flushed with N_2_ were used. The values shown represent the mean ± standard deviation (*n*=3).

Cathode	−5 mA, 7 d	−20 mA, 7 d	−40 mA, 7 d	−60 mA, 7 d	−80 mA, 7 d	
Carbon rod	0.40 ± 0.03	1.29 ± 0.04	1.61 ± 0.50	2.45 ± 1.31a)	4.94b)	Acetate [g L^−1^]
Carbon fiber fabric	0.19 ± 0.02	1.29 ± 0.12	3.32 ± 0.21	6.08 ± 0.96	9.28 ± 2.10	
Carbon rod	15.9 ± 1.0	17.9 ± 0.3	21.1 ± 0.5	18.2 ± 4.0a)	14.5b)	Glycine [mg L^−1^]
Carbon fiber fabric	15.2 ± 0.8	17.0 ± 1.8	17.8 ± 5.4	17.6 ± 9.2	17.1 ± 11.1	
Carbon rod	1.6 ± 1.1	52.6 ± 4.5	204.1 ± 83.0	233.5 ± 121.7a)	364.0b)	Ethanol‐amine [mg L^−1^]
Carbon fiber fabric	0.7 ± 0.2	51.6 ± 5.2	215.8 ± 31.6	263.9 ± 32.8	284.5 ± 64.4	

a) *n* = 2.

b) *n* = 1.

Increasing the current led not only to higher acetate production but also to an increase in maximal OD_600 nm_ to 0.298 (*n* = 1) for the carbon rod as well as 0.265 ± 0.038 for the carbon fiber fabric cathode electrobioreactors (Figure S4, Supporting Information), indicating a better growth of *C. ljungdahlii* due to higher H_2_ availability.

Glycine and ethanolamine concentrations, again, did not differ between the two different cathode materials. The initial glycine concentration of 14.5 ± 0.7 mg L^−1^ showed no important changes over time (Table 4). The maximal titer of 21.1 ± 0.5 mg L^−1^ was reached after 21 days of operation with carbon rod cathodes. In the electrobioreactor setups with carbon fiber fabric cathodes, a slight increase of glycine concentration to 17.0 ± 1.8 mg L^−1^ was recorded after 14 days, but no further increase followed. In contrast, the ethanolamine concentration continuously increased and reached a final concentration of 284.5 ± 64.4 mg L^−1^ for MES from CO_2_ in electrobioreactors with a carbon fiber fabric cathode and 364.0 mg L^−1^ (*n* = 1) for carbon rod cathode electrobioreactors. Unlike for acetate levels, glycine and ethanolamine concentrations did not appear to differ between the two cathode materials (however, no full evaluation is possible, since only one carbon rod system persisted to the end). Thus, higher H_2_ availability does not seem to affect production thereof. This raises the question of whether production of glycine and ethanolamine is a stress response to the overall reaction setup, e.g., the applied current, rather than a consequence of H_2_ limitation.

Regarding the CEs (**Table** **5**), we observed a clear initial performance deficit for carbon fiber fabric cathodes while operating at −5 mA (being in line with the first experiments, Table 3) when compared to carbon rod cathode electrobioreactors. Again, we expect a lack of accuracy for the first few days, when yeast extract was still available as a second carbon and energy source. This might explain the unrealistically high CE of 139.1 ± 8.5% observed for the carbon rod cathodes during the operation with a current of −5 mA. However, this does not explain the low CE of 52.7 ± 4.0% of the carbon fiber fabric cathode electrobioreactors. An initially low CE points to a severe loss of reducing equivalents by H_2,_ leaving the reactor system unused, as can be expected when the biomass concentration is still low. Leaving this initial phase aside for a deeper analysis, the highest CEs were observed for both electrobioreactor configurations at −20 mA (76.8 ± 0.7% and 103.8 ± 10.1% for the carbon rod and carbon fiber fabric cathode, respectively). Remarkably, this high CE dropped to a low level of around 20% CE for all higher currents for the carbon rod electrode. This shows that a local input of the electrical energy on fairly small electrode surfaces results in a strong local production of H_2_, which mostly remains unused and is flushed out with the reactor gassing. Furthermore, this localized high energy input may result in mechanical stress to the anode and cathode and heat dissipation, both resulting in the disintegration of the electrode material, evaporation, and hence abortion of two of the three systems. What is also noticeable for the carbon rod cathode electrobioreactors is that the biomass growth considering the OD_600 nm_ (Figure S4, Supporting Information) stagnated at an applied potential of −20 mA. For electrobioreactors using carbon fiber fabric cathodes, the CE dropped from 103.8 ± 10.1% at −20 mA to 93.4 ± 12.2% at −40 mA, 74.4 ± 20.7% at −60 mA, and 59.2 ± 22.0% at −80 mA, with increasing standard deviation. Thus, the distribution of H_2_ evolution throughout the electrobioreactor volume warrants a more efficient biocatalytic utilization to form acetate, resulting in lower losses of H_2_. But while this indeed also leads to continuous biomass growth until −60 mA (based on OD_600nm_), the rate of H_2_ input is higher than the rate of biocatalyst build‐up resulting in a decrease of CE at higher currents. Considering the CE of the complete experiment, the same trend was recorded as before, with a distinct higher CE of 79.7 ± 18.0% for carbon fiber fabric cathode electrobioreactors compared to carbon rod electrobioreactors with a CE of 32.7 ± 10.8%.

**Table 5:** The CE of the MES from CO_2_ using *C. ljungdahlii* for the different electrobioreactor setups under galvanostatic operation. The electrobioreactors were operated for 35 days with a stepwise current increase from −5 to −20, −40, −60, and −80 mA every 7 days. Two types of cathodes, carbon rod cathodes and carbon fiber fabric cathode, in combination with an OER anode flushed with N_2_ were used. The calculation of CE includes the formation of acetate, glycine and ethanolamine, and values shown represent the mean ± standard deviation (*n* = 3).

Cathode	CE [%]					
	Complete, 35 d	−5 mA, 7 d	−20 mA, 7 d	−40 mA, 7 d	−60 mA, 7 d	−80 mA, 7 d
Carbon rod	32.7 ± 10.8	139.1 ± 8.5	76.8 ± 0.7	18.8 ± 24.0	21.8 ± 20.5a)	12.1b)
Carbon fiber fabric	79.7 ± 18.0	52.7 ± 4.0	103.8 ± 10.1	93.4 ± 12.2	74.4 ± 20.7	59.2 ± 22.0

a) *n*=2.

b) *n* = 1.

With the stepwise increase in the current regime up to −80 mA, it is shown that *C. ljungdahlii* can resist such a high current flow, and the MES performance increased remarkably. As the acetate concentration from MES still increased linearly with the current, we aimed to evaluate the maximum performance by getting into a steady‐state. Therefore, a third experiment with a faster stepwise current increase starting from −20 mA for 3 days to −60 mA for a further 4 days and finally to −80 mA for 24 days was applied to the identical electrobioreactor setups. We considered that the fast increase in current might come at the cost of CE, since biomass growth may not follow fast enough. At the same time, we hypothesize that CE should recover by an extended time at the high current, where biomass should build up. To avoid limitations of performance by organic acid toxicity to the biocatalyst by product accumulation, we kept the pH between 5.7 and 5.2 that is well above pH 4.8 that was shown to be suitable for growth.^[^ ^19^, ^20^ ^]^

**Figure 5:**
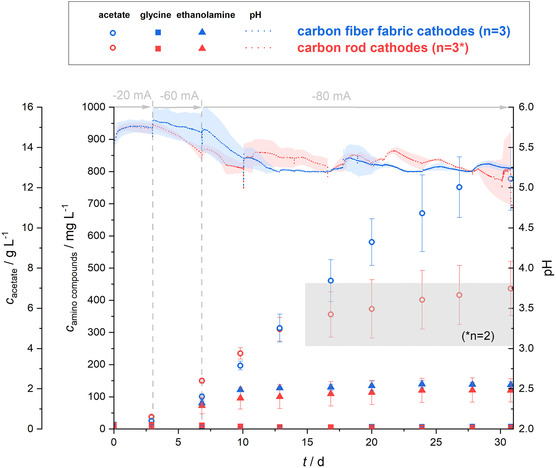
MES from CO_2_ using *C. ljungdahlii*: The figure illustrates the concentration of acetate and of the amino compounds glycine and ethanolamine as well as the development of pH in electrobioreactors under galvanostatic operation. The electrobioreactors were operated for 31 days with a stepwise current increase from −20 mA (3 d) to −60 mA (4 d) and −80 mA (24 d). Two types of cathodes, carbon rod cathodes (red) and carbon fiber fabric cathode (blue), in combination with an OER anode flushed with N_2_ were used. The error bars and shaded areas represent the standard deviation of the mean (*n* = 3). Initially, each electrobioreactor setup was started in triplicates. However, the carbon rod system failed for one replicates during the experiment (indicated by *n = 2 in the grey box).

In the initial phase (**Figure** **5**), using carbon rod cathodes showed a better performance in terms of acetate production compared to carbon fiber fabric cathodes, specifically 2.39 ± 0.12 g L^−1^ compared to 1.61 ± 0.15 g L^−1^ after 7 days. However, an increase of current to −80 mA showed the benefits of a well‐distributed electric current and hence H_2_ input to the biocatalytic process and the expected better performance of acetate production by carbon fiber fabric cathodes in electrobioreactors (**Table** **6**). On day 13 (being 5 days at −80 mA), the acetate concentrations were in the same range in both electrobioreactor types (around 5 g L^−1^). After 31 days of operation and thus 24 days at −80 mA, a final acetate concentration of 6.98 ± 1.35 g L^−1^ for carbon rod cathodes and 12.44 ± 1.56 g L^−1^ for carbon fiber fabric cathodes was achieved. Maximal OD_600 nm_ was reached with 0.396 ± 0.018 for the carbon rod and 0.555 ± 0.058 for the carbon fiber fabric cathode electrobioreactors (Figure S5, Supporting Information), showing the benefit of the distributed H_2_ input for biomass development. This does not even consider that it is likely that the larger surface‐area electrode also housed more bound biomass that was not quantified at this stage. With the combination of a controllable electrobioreactor setup, a large‐surface‐area carbon fiber fabric cathode combined with OER anodes flushed with N_2_, and the chosen current regime, we present the highest reproducible acetate concentration of 12.44 ± 1.56 g L^−1^ being reported in MES from CO_2_ using a pure culture. Similar values were only achieved by Bajracharya et al. (2022)^[^ ^11^ ^]^ reporting ≈11 g L^−1^ acetate (*n* = 1) during 34 days operation MES from CO_2_ with *Sporomusa ovata* under potentiostatic control of −0.9 V vs Ag/AgCl in H‐cell type reactors with dual cathode of carbon rod and titanium mesh and headspace recirculation.

**Table 6:** The concentration of acetate as a main product of the MES from CO_2_ using *C. ljungdahlii* as well as the amino compounds glycine and ethanolamine. The concentrations are presented the different electrobioreactor setups under galvanostatic operation. The electrobioreactors were operated for 31 days with a stepwise current increase from −20 mA (3 d) to −60 mA (4 d), and −80 mA (24 d). Two types of cathodes, carbon rod cathodes and carbon fiber fabric cathode, in combination with an OER anode flushed with N_2_ were used. The values shown represent the mean ± standard deviation (*n* = 3).

Cathode	−20 mA, 3 d	−60 mA, 4 d	−80 mA, 24 d	
Carbon rod	0.60 ± 0.07	2.39 ± 0.12	6.98 ± 1.35	Acetate [g L^−1^]
Carbon fiber fabric	0.40 ± 0.06	1.61 ± 0.15	12.44 ± 1.56	
Carbon rod	14.3 ± 1.2	10.0 ± 1.8	7.4 ± 0.1	Glycine [mg L^−1^]
Carbon fiber fabric	12.9 ± 2.8	11.4 ± 2.7	4.5 ± 1.3	
Carbon rod	1.8 ± 0.5	80.8 ± 6.1	136.9 ± 9.7	Ethanolamine [mg L^−1^]
Carbon fiber fabric	2.1 ± 0.2	72.6 ± 25.6	120.3 ± 36.3	

Again, production of glycine was negligible, and levels of ethanolamine increased independently of the used cathode material, but stagnated around day 13 at 120 mg L^−1^ for both setups without further increase. CEs (**Table** **7**) again decreased when applying higher currents from 137.6 ± 19.2% at −20 mA to 103.8 ± 3.0% at −60 mA and 34.5 ± 9.8% at −80 mA for the carbon rod and 98.4 ± 17.1% at −20 mA to 70.3 ± 10.1% at −60 mA and 67.5 ± 9.9% at −80 mA for carbon fiber fabric cathode electrobioreactors, respectively. The overall CE of 67.7 ± 8.5% of carbon fiber fabric cathode electrobioreactors compared to 46.2 ± 12.0% equipped with carbon rod cathodes showed a clear advantage of using cathodes with an improved spatial H_2_ distribution. Even for the highest applied current (−80 mA), independent of material, the cathode potential was not more negative than ≈−1.2 V vs Ag/AgCl sat. KCl (Table S1, Supporting Information), which clearly indicates a low energetic burden of MES. Unlike what we suspected, the fast increase in current did not lead to a severe loss of reducing equivalents due to a lack of matching biomass and biocatalytic activity. Instead, the growth rate of *C. ljungdahlii* for the first 7 days of experiment was drastically increased from 0.009 ± 0.001 h^−1^ and 0.004 ± 0.002 h^−1^ at −5 mA (Figure S4, Supporting Information) to 0.043 ± 0.001 h^−1^ and 0.024 ± 0.002 h^−1^ at−20 mA and−60 mA (Figure S5, Supporting Information) for carbon rod and carbon fiber fabric cathodes, respectively, with the higher H_2_ availability. We strongly assume that this is due to the improved H_2_ distribution, facilitated by the novel 3D‐architecture of the carbon fiber fabric electrode. The specific increase of the H_2_ availability by the larger active surface area for electrochemical H_2_ formation and how it supports microorganisms for a more efficient biocatalytic conversion of H_2_ to acetate needs further investigation, e.g., determination of the kLa‐value or modeling.

**Table 7:** The CE of the MES from CO_2_ using *C. ljungdahlii* for the different electrobioreactor setups under galvanostatic operation. The electrobioreactors were operated for 31 days with a stepwise current increase from −20 mA (3 d) to −60 mA (4 d) and −80 mA (24 d). Two types of cathodes, carbon rod cathodes and carbon fiber fabric cathode, in combination with an OER anode flushed with N_2_ were used. The calculation of CE includes the formation of acetate, glycine and ethanolamine, and the values shown represent the mean ± standard deviation (*n* = 3).

Cathode	CE [%]			
	Complete, 31 d	−20 mA 3 d	−60 m, 4 d	−80 mA, 24 d
Carbon rod	46.2 ± 12.0	137.6 ± 19.2	103.8 ± 3.0	34.5 ± 9.8
Carbon fiber fabric	67.7 ± 8.5	98.4 ± 17.1	70.3 ± 10.1	67.5 ± 9.9

With a maximum acetate production rate of the whole operation period of 0.4 ± 0.05 g L^−1^ d^−1^ and a final acetate titer of 12.44 ± 1.56 g L^−1^ using carbon fiber fabric cathode electrobioreactors with a surface‐area‐to‐reactor‐volume ratio of 166.7 cm^2^ L^−1^, we achieved the highest ever reported performance for MES from CO_2_ using *C. ljungdahlii*. With regard to the phase of maximum productivity from day 10 to day 20 (i.e., linear increase of acetate), the maximum acetate production rate is even 0.6 ± 0.1 g L^−1^ d^−1^. Compared to gas fermentation with *C. ljungdahlii* with a maximum reported rate for acetate production of 2.00 g L^−1^ d^−1^^[^ ^21^ ^]^ as well as MES from CO_2_ with mixed cultures with ≈1.9 g L^−1^ d^−1^ (14 studies, Harnisch et al. 2024), MES has now reached ≈30% of their performance. This is a great improvement compared to previous studies of MES by pure cultures with only ≈0.087 g L^−1^ d^−1^ acetate production rate (16 studies, Harnisch et al. 2024), or specifically for *C. jungdahlii* with 0.11 g L^−1^ d^−1^,^[^ ^9^ ^]^ and shows that the strategy of matching electrobioreactor design to the needs of the biocatalyst is the right way to move toward success of electrobiotechnology.^[^ ^22^ ^]^ Strategies to advance MES toward application include further enhancements to the electrobioreactor design. These include, for example, the development of alternative anode reactions, which will be crucial for upscaling in particular, as continuous N_2_ flushing on a large scale is both unsustainable and costly. Additionally, optimized H_2_ recycling is essential to increase the overall efficiency of the MES process, enabling consistent H_2_ availability with reduced energy input. Moreover, addressing mass transfer limitations in the current electrobioreactor design, caused by insufficient mixing of the system, can be achieved by improving the stirrer design.^[^ ^18^ ^]^

## 3 Conclusion

MES from CO_2_ using *C. ljungdahlii*, a strictly anaerobic model acetogen, benefits substantially from an optimized electrobioreactor environment. Our results clearly show that a higher MES performance is based on the decrease of oxidative stress that is mainly caused by the O_2_ crossover from the anode chamber. Here, N_2_ flushing is an efficient method to remove O_2_ on a small scale, but scaling up is associated with unsustainability and higher costs. Thus, efficient alternative anodic reactions for upscaled electrobioreactors need to be developed. In addition to reducing O_2_ stress, MES performance can benefit from improved H_2_ availability, which is achieved by increasing the current up to −80 mA to boost the H_2_ production, as well as using a carbon fiber fabric cathode that improves spatial H_2_ distribution and thus availability. The decreased CE at even more negative currents indicates that H_2_ availability can only improve MES performance to a certain limit; after that, the H_2_ will be lost through outgassing. Further studies on strategies to prevent the loss of produced H_2_ are urgently needed and of high relevance. Possible measures include, for example, optimized recycling of the H_2_, improving the distribution, as well as using pressurized electrobioreactors to ensure higher efficiency with lower energy input.

Our results also indicate no link between H_2_ availability and the production of glycine and ethanolamine. Those two products are produced by *C. ljungdahlii* only during MES, which raises the question of whether their production is a stress response to the applied current rather than a consequence of H_2_ limitation.

## 4 Experimental Section

4.1

4.1.1

##### Chemicals

All used chemicals were of at least analytical grade and were supplied from Carl Roth GmbH (Germany) and Merck KGaA (Germany). The used gases had a purity of not less than 99.8% and were supplied by Air Products GmbH (Germany). Deionized water (Merck KGaA, Germany) was used to prepare all microbial growth media.

##### Bacterial Strain and Cultivation

*C. ljungdahlii* DSM 13 528 (DSMZ) was cultured heterotrophically in reinforced Clostridia media (RCM) (10 g L^−1^ peptone, 10 g L^−1^ meat extract, 3 g L^−1^ yeast extract, 5 g L^−1^ NaCl, 5 g L^−1^ fructose, 1 g L^−1^ soluble starch, 5 g L^−1^ Na‐acetate × 3H_2_O, 0.5 g L^−1^ l‐cysteine × HCl, 1 mg L^−1^ resazurin). Autotrophic and electroautotrophic cultivation was performed in modified PETC media (20 g L^−1^ 2‐(*N*‐morpholino)ethanesulfonic acid, 2.0 g L^−1^ NH_4_Cl, 0.2 g L^−1^ KCl, 0.2 g L^−1^ NaCl, 0.2 g L^−1^ KH_2_PO_4_, 0.4 g L^−1^ MgSO_4_ × 7H_2_O, 80 mg L^−1^ CaCl_2_ × 2H_2_O, 2 mg L^−1^ CoCl_2_ × 6H_2_O, 8 mg L^−1^ (NH_4_)_2_Fe(SO_4_)_2_, 10 mg L^−1^ MnSO_4_, 20 mg L^−1^ nitrilotriacetic acid, 2 mg L^−1^ Na_2_MoO_4_, 0.2 mg L^−1^ Na_2_SeO_4_, 0.2 mg L^−1^ Na_2_WO_4_, 2 mg L^−1^ ZnSO_4_, 0.02 mg L^−1^ biotin, 0.05 mg L^−1^ pantothenic acid, 0.05 mg L^−1^ thiamine HCl, 1 g L^−1^ yeast extract, 0.3 g L^−1^ l‐cysteine HCl, 1 mg L^−1^ resazurin) adjusted to pH 5.7.

Heterotrophic pre‐cultures of *C. ljungdahlii* were grown in 10 mL of RCM in Hungate tubes in serial transfers (2 transfers, each 24 h of incubation for total of 48 h precultivation). Then, a final transfer to 30 mL of PETC in 250 mL serum bottles was performed to facilitate acclimation to autotrophic conditions. The serum bottles were tightly closed with butyl rubber stoppers, the headspace was filled with 80/20 v/v H_2_/CO_2_ at an overpressure of 1.5 bar and incubated horizontally for 6–7 days before serving as inoculum for the electrobioreactors. All cultivations were performed at 37 °C under strict anaerobic conditions using an anaerobic working station (Whitley A 135 HEPA, Meintrup DWS Laborgeräte GmbH, Germany) for the media preparation and microbial transfers. All transfers were made using 10% v/v of the media to be inoculated.

##### Electrobioreactor Design and Operation: Electrobioreactor and Electrode Setup

The MES from CO_2_ using *C. ljungdahlii* was carried out in a conventional 1‐L glass bioreactor CSTR system (Multifors, Infors HT, Switzerland) in combination with a bioelectrochemical upgrade kit^[^ ^12^, ^13^ ^]^ allowing to place cathodes as working electrodes (WE) in the main vessel and anodes as counter electrodes (CE) in a chamber designed as a central inlay (Figure 1A). WE and CE of the upgrade kit originally consist of two pairs of carbon rods (CP‐Handels GmbH, Germany) with a surface area of 35.3 cm^2^ each. An Ag/AgCl sat. KCl reference electrode (+ 0.197 V vs SHE, SE 11, Xylem Analytics Germany Sales GmbH & Co. KG Sensortechnik Meinsberg, Germany) was used. The WE and CE chambers were separated by a cation exchange membrane (Fumasep FKE‐50, Fumatech BWT GmbH, Germany). The cathode compartment was filled with 850 mL PETC media, whereas the anode compartment was filled with PETC 150 mL media. For the different electrobioreactor setups, different anodes and cathodes were used (Table 1, Figure 1B,C). As an alternative to carbon rod cathodes, a carbon fiber fabric material (T300, *w* = 4 mm, Güth & Wolf GmbH, Germany, described in Pötschke et al. 2022) was used as cathode with a geometric surface area of 144 cm^2^ fixed in a 3D‐printed resin holder (Figure S6, Supporting Information; BioMed Clear Resin, Formlabs GmbH, Germany). A titanium wire (0.5 mm diameter, Goodfellow GmbH, Germany) being manually interwoven was used as current collector. As an alternative material to the carbon rod anode leading to OER, a carbon felt (Sigracell battery felt GFD 4.65 EA, Germany) with a surface area of 45 cm^2^ was used. The carbon felt acted as a sacrificial anode (similar to that described in Rohbohm et al. 2023)^[^ ^14^ ^]^ and was activated prior to use by applying a current of 20 A m^−2^ for 12 h in 1 M sodium hydroxide solution.

##### Preparation and Operation of Electrobioreactors

Electrobioreactors including a major part of the upgrade kit (except of RE, CE chamber with membrane) and the basic microbial growth media (without 2‐(*N*‐morpholino)ethanesulfonic acid, vitamins, and trace elements) were autoclaved before each experiment. The reference electrodes were chemically sterilized in Beckmann solution (70% ethanol, 0.1 M H_2_SO_4_) for at least 30 min and rinsed with sterile water. The CE chamber was autoclaved separately under dry conditions to avoid damage to the membrane. When using the activated carbon felt as sacrificial anode, the material was rinsed with sterile water after activation. For the N_2_ flushing of the CE chamber, a needle (2.00/1.50 × 200 mm) was installed reaching the bottom. To avoid overpressure in the CE chamber while flushing and causing membrane damages a gas outlet was installed. After autoclaving and cooling down the electrobioreactors, all missing media components were sterile filtered and added, and all remaining parts of the upgrade kit were installed in a sterile workstation while the reactor headspace was purged with N_2_. The electrobioreactors were inoculated with 50 to 60 mL of autotrophic *C. ljungdahlii* pre‐culture. A continuous purging of the cathode chamber with a gas mixture of 80/20 v/v N_2_/CO_2_ with a flow rate of 0.35 L min^−1^ was performed to ensure anaerobic conditions and the supply with the carbon source for autotrophic growth. All experiments were performed at 37 °C and stirred at 200 rpm. The pH value was either only recorded or controlled by adding 20% sodium hydroxide v/v after the initial pH dropped from 5.7 to 5.2. The process parameters of the electrobioreactors (stirring, gassing, temperature, and pH control) were monitored and controlled via eve software (Infors HT, Switzerland). Always six reactors were run in parallel with two conditions and three independent replicates each. All experiments were carried out galvanostatically, i.e., under chronopotentiometric control, using a multichannel and a single‐channel potentiostat/galvanostat (VSP and SP 200, BioLogic Science Instruments, France). The applied current at the cathode was set to −5, −20, −40, −60, or −80 mA (resulting potentials see Table S1, Supporting Information) as detailed below for the different experimental setups. After each electrobioreactor experiment membrane integrity was checked visually as well as by assuring to be leakage free after operation.

##### Analytics

High performance liquid chromatography (HPLC) analyses were performed to determine the concentration of the organic acid acetate as well as amino compounds like glycine and ethanolamine. Acetate was measured using a HPLC system (Shimadzu Scientific Instruments, Japan) equipped with a photodiode array and a HiPlex H 300 × 7.7 mm column (Agilent Technologies, Inc. CA, USA) with a precolumn SecurityGuard Cartridge Carbo‐H (4 × 3.0 mm ID, Phenomenex, USA). The mobile phase used was 0.1 N H_2_SO_4_. The samples were run for 30 min in isocratic mode with a flow rate of 0.5 mL min^−1^ at 50 °C. Calibration and peak identification were carried out with external standards (four‐point calibration from ≈0.1 g L^−1^ to ≈1 g L^−1^, *R* ^2^ = 0.999).

The concentration of the amino compounds ethanolamine and glycine was measured using another HPLC system (Jasco Corporation, Japan). Derivatization was done by mixing 96 μL of the sample with 96 μL of the derivatizing agent *o*‐phthaldialdehyde, and the reaction was allowed to proceed for 2 min.^[^ ^9^ ^]^ 1 μL of the sample was injected into the HPLC system with a UV detector (338 nm) and a fluorescence detector (excitation: 340 nm; emission: 455 nm). For separation, Kinetex XB C18 column (100 mm × 2.1 mm ID, 2.6 μm; Phenomenex, USA) was paired with a SecurityGuard Ultra Cartridge C18 (2.1 mm ID; Phenomenex, USA) at 25 °C. Gradient elution (Table S2, Supporting Information) was done using mobile phase A (20 mM potassium phosphate buffer, pH 7.2) and mobile phase B (acetonitrile: methanol, 50:50 v/v, with a flow rate of 0.25 mL min^−1^.

##### TEM

*C. ljungdahlii* cell suspensions (10–15 mL) were centrifuged at 8.000 × *g* for 10 min, and the resulting cell pellets were chemically fixed by adding 2 mL of 2.5% glutaraldehyde in sodium cacodylate buffer (pH 7.2, 0.1 M) for 1 h at room temperature. After further centrifugation, the pellets were washed twice with pure sodium cacodylate buffer (0.1 M, pH 7.2) and stored at 4 °C until analysis. Cell pellets were postfixed with 1% osmium tetroxide in cacodylate buffer for another 2 h, followed by an ascending ethanol series. Cell samples were embedded in Araldite resin (Plano, Germany) according to the manufacturer's instructions. Ultrathin sections (thickness 50 nm) were cut using an Ultracut E ultramicrotome (Reichert‐Jung, Austria) and applied onto Formvar/Carbon coated copper grids (100 meshes, Quantifoil Micro Tools, Germany). The sections were stained for 10 min with lead citrate and then examined in a digitally upgraded Point Electronic Zeiss EM900 electron microscope (Point Electronic, Germany; Zeiss, Germany) operated at 80 kV. Digitized images were taken with a wide‐angle dual‐speed 2 K CCD camera controlled by a Sharp/Eye base controller and operated by the Image SP software (camera and software TRS, Germany).

##### Data Analysis and Statistics

CE, which describes the overall electron efficiency of the MES from CO_2_, was calculated according to Equation (1).

(1) $$\text{CE}=\frac{\Delta C\times V\times z\times F}{\int_{0}^{t}Idt}\times 100$$

where ΔC denotes the change of product concentration in mol L^−1^, *V* denotes the volume in L, *z* denotes the number of electrons (8 for reduction of CO_2_ to acetate and 6 for reduction to glycine and ethanolamine), *F* denotes the Faraday constant of 96485.3 C mol^−1^, I denotes the current in A, and *t* denotes the time in s.

All experiments were conducted in triplicates and values are provided as mean with standard deviation.

## Conflict of Interest

The authors declare no conflict of interest.

## Supporting information

Supplementary Material

## Data Availability

The data that support the findings of this study are available in the supplementary material of this article.
