# Supplementary material for: Reducing Oxygen Stress and Improving Hydrogen Availability Boosts Microbial Electrosynthesis by Clostridium ljungdahlii
Source: ChemSusChem. 2025 Sep 11;18(21):e202501118. doi: 10.1002/cssc.202501118 (PMC12584966; doi:10.1002/cssc.202501118)
Supplement: Supplementary file 1 — Supplementary Material [file CSSC-18-e202501118-s001.zip › 3_Supporting Information_upload_for_production.docx]

Supplementary Material

**Reducing Oxygen Stress and Improving Hydrogen Availability Boosts Microbial Electrosynthesis by *Clostridium ljungdahlii***

Anne Kuchenbuch ^[a]^, Sara Al-Sbei ^[b,c]^, Luis F. M. Rosa ^[a],[$]^, Santiago T. Boto ^[b]^, Martin Westermann ^[d]^, Miriam A. Rosenbaum ^[b,c]^ and Falk Harnisch *^[a]^

[a] A. Kuchenbuch, Dr. L. F. M. Rosa, Prof. F. Harnisch

Department of Microbial Biotechnology

Helmholtz-Centre for Environmental Research GmbH – UFZ

Permoserstr. 15, 04318 Leipzig, Germany

E-mail: falk.harnisch@ufz.de

[b] S. Al-Sbei, Dr. S.T. Boto, Prof. M. Rosenbaum

Department Bio Pilot Plant
Leibniz Institute for Natural Product Research and Infection Biology – Hans-Knöll Institute

Beutenbergstr. 11a, 07745 Jena, Germany

[c] S. Al-Sbei, Prof. M. Rosenbaum

Faculty of Biological Science

Friedrich Schiller University Jena

Bachstr. 18k, 07743 Jena, Germany

[d] Dr. M. Westermann

Electron Microscopy Center

Jena University Hospital

Ziegelmühlenweg 1

07743 Jena

Germany

[$] current affiliation: Dr. L. F. M. Rosa

Biological and Environmental Science and Engineering Division (BESE)

King Abdullah University for Science and Technology (KAUST)

Thuwal, 23955-6900 Saudi Arabia

**SI 1: Supplementary results**

**SI 1.1: The use of a cation exchange membrane is insufficient to prevent oxygen crossover during MES**

Materials and methods: Two different cation exchange membranes were tested in the electrobioreactors to shield the cathode chamber from the O_2_ produced by the anodic reaction: Fumasep FKE-50 membrane (Fumatech BWT GmbH, Germany, thickness 50 µm) and CMI-7000S cation exchange membrane (Membranes International, USA, thickness 450 µm). The membranes were cut to a size of 98 × 67 cm^2^ and glued using Loctite EA 3430 glue (Henkel AG & Co. KGaA, Germany) into the window between the chambers. After curing overnight, the reactors were assembled and autoclaved under dry conditions. Sterile PETC media was added immediately before testing. To establish anaerobic conditions, N_2_ was flushed continuously for two hours before starting the test. The WE and CE each consisted of two carbon rods, as described in section 1.3.1, and an Ag/AgCl saturated KCl reference electrode was connected to the potentiostat. A potential of -0.9 V vs. Ag/AgCl was applied. The reactors were operated abiotically under continuous nitrogen flushing. After approximately 19 h, nitrogen flushing was stopped to evaluate oxygen crossover resulting from anodic oxygen evolution from water electrolysis. Dissolved oxygen levels were monitored using optical dissolved oxygen sensors (VisiFerm DO Arc 225, Hamilton, Switzerland), which were calibrated beforehand in a two-point calibration with an anaerobic PETC media and an overnight air-flushed PETC media. Oxygen measurement was conducted only in the cathode chamber as it was not possible in the anode chamber inlay due to geometric limitations.

Results: We tested two different membranes to evaluate their ability to prevent the crossover of the oxygen produced by the anodic oxygen evolution reaction (OER) to the cathode chamber. In the end, no membrane completely isolated the cathode chamber from oxygen without nitrogen flushing, and the oxygen level started to increase immediately after nitrogen flushing was switched off to reach about the same level for both membranes (SI Figure1). However, we proceeded with the Fumasep FKE-50 membrane, as it offers lower electric resistance and more mechanical flexibility.


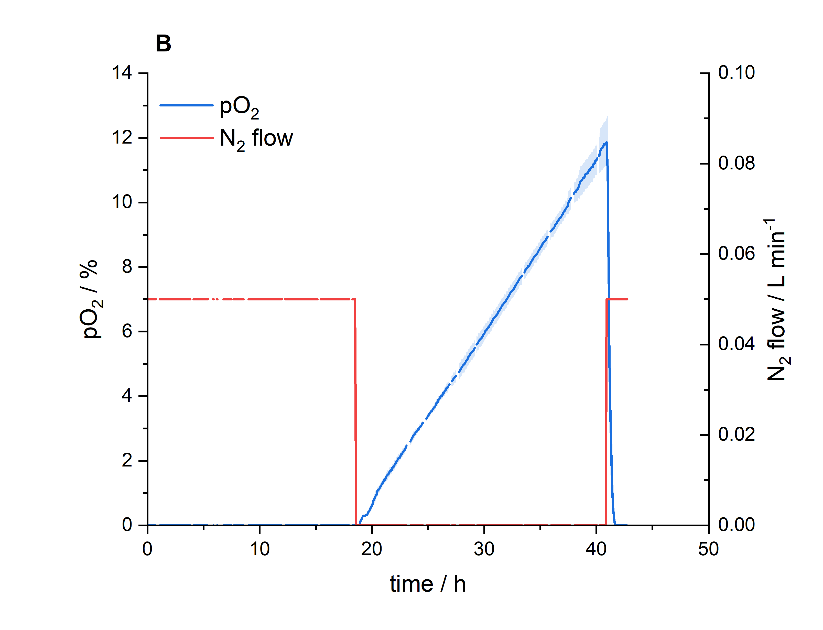

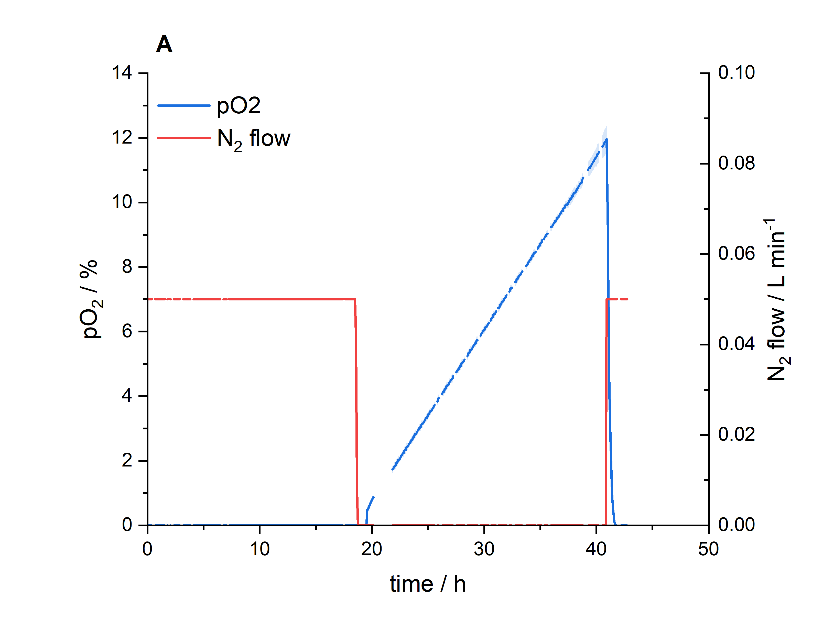


**SI Figure 1:** Oxygen levels in the cathode chamber of the electrobioreactor using two membranes: (A) Fumasep FKE-50 and (B) Membrane International CMI-7000S. Oxygen produced by the OER at the anode reached the cathode chamber when nitrogen flushing was stopped. Both membranes showed similar results; no oxygen was detected with continuous nitrogen flushing, but oxygen gradually increased in the cathode chamber once flushing ceased. Oxygen measurement on the anode side was not possible due to geometric limitations. (n=3)

**SI 1.2: Microbial growth during microbial electrosynthesis from CO_2_ of *C. ljungdahlii* in electrobioreactors**


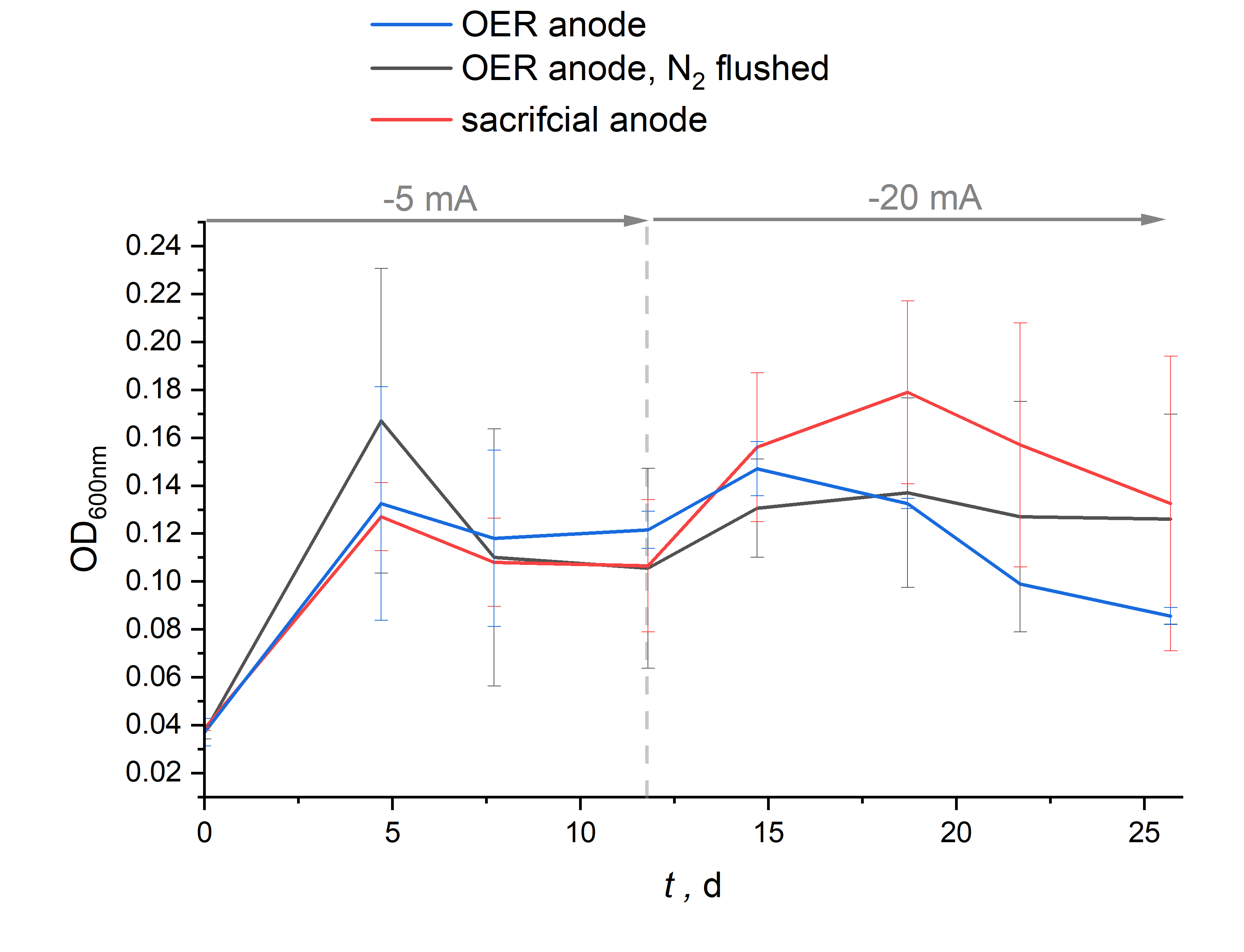


**SI Figure 2:** Microbial electrosynthesis from CO_2_ using *C. ljungdahlii*: OD_600nm_ in electrobioreactors under galvanostatic operation. The electrobioreactors were operated for 12 d at a current of -5 mA and further 14 d at -20 mA. Carbon rod cathodes in combination with OER anode (blue), OER anode flushed with N_2_ (black) and sacrificial anode (red) were used.


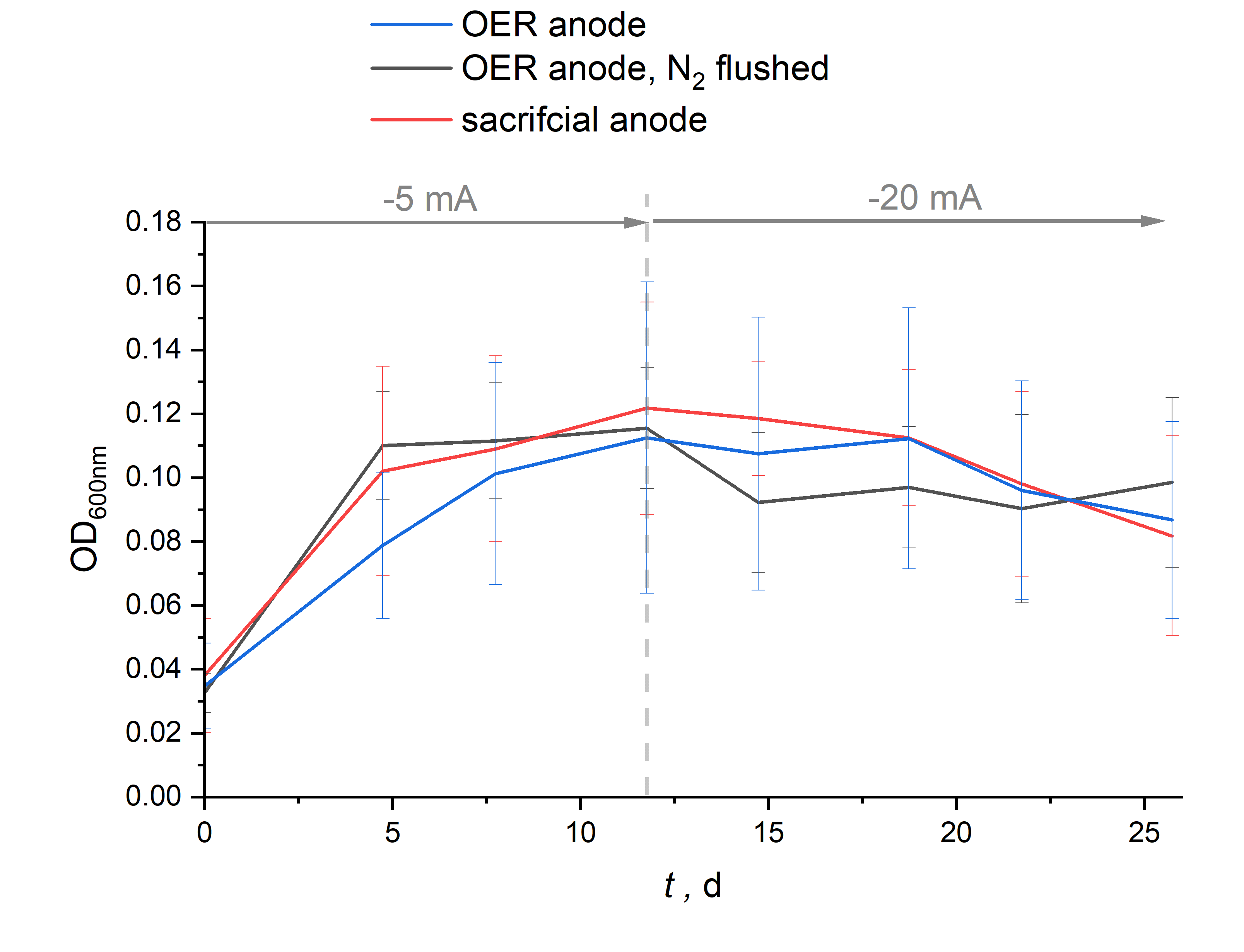


**SI Figure 3:** Microbial electrosynthesis from CO_2_ using *C. ljungdahlii*: OD_600nm_ in electrobioreactors under galvanostatic operation. The electrobioreactors were operated for 12 d at a current of -5 mA and further 14 d at -20 mA. Carbon fiber fabric cathodes in combination with OER anode (blue), OER anode flushed with N_2_ (black) and sacrificial anode (red) were used.


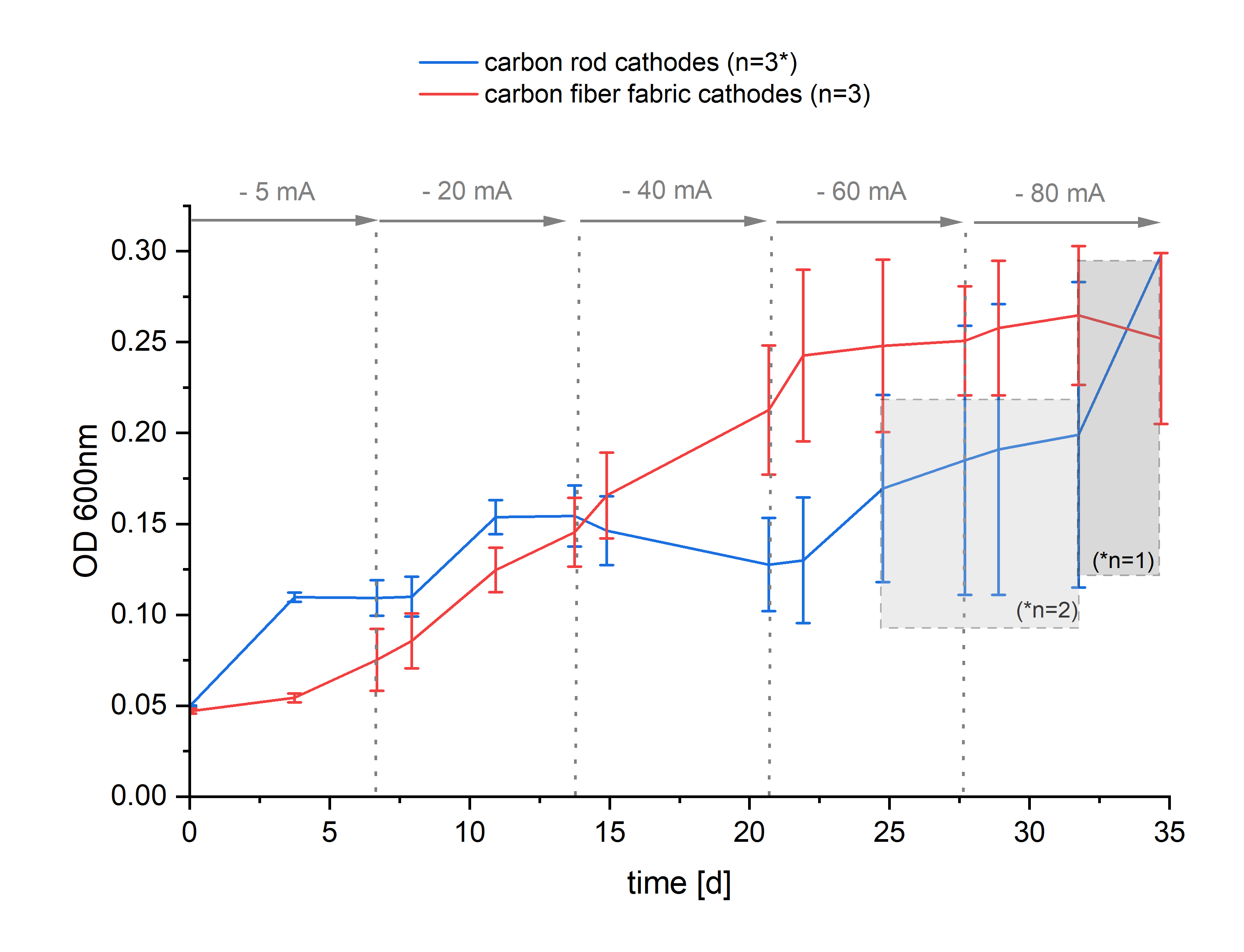


**SI Figure 4:** Microbial electrosynthesis from CO_2_ using *C. ljungdahlii*: OD_600nm_ in electrobioreactors under galvanostatic operation. The electrobioreactors were operated for 35 d with a stepwise current increase from -5 mA to -20 mA, -40 mA, -60 mA and -80 mA every seven days. Two cathode types, carbon rod cathodes (blue) and carbon fiber fabric cathode (red), in combination with OER anode flushed with N_2_ were used.


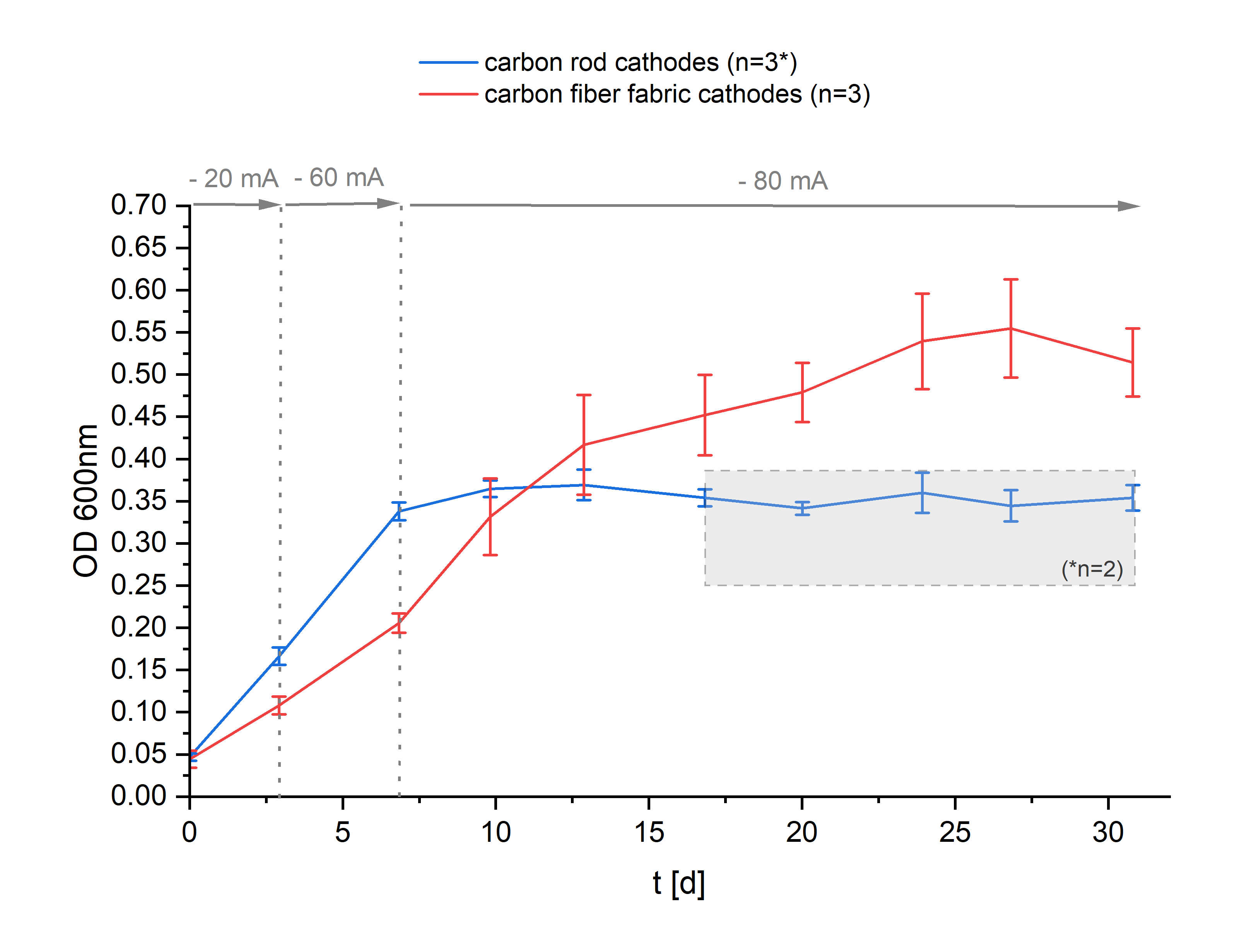


**SI Figure 5:** Microbial electrosynthesis from CO_2_ using *C. ljungdahlii*: OD_600nm_ in electrobioreactors under galvanostatic operation. The electrobioreactors were operated for 31 d with a stepwise current increase from -20 mA (3 d) to -60 mA (4 d) and -80 mA (24 d). Two cathode types, carbon rod cathodes (blue) and carbon fiber fabric cathode (red), in combination with OER anode flushed with N_2_ were used.

**SI 2: Supplementary materials and methods**


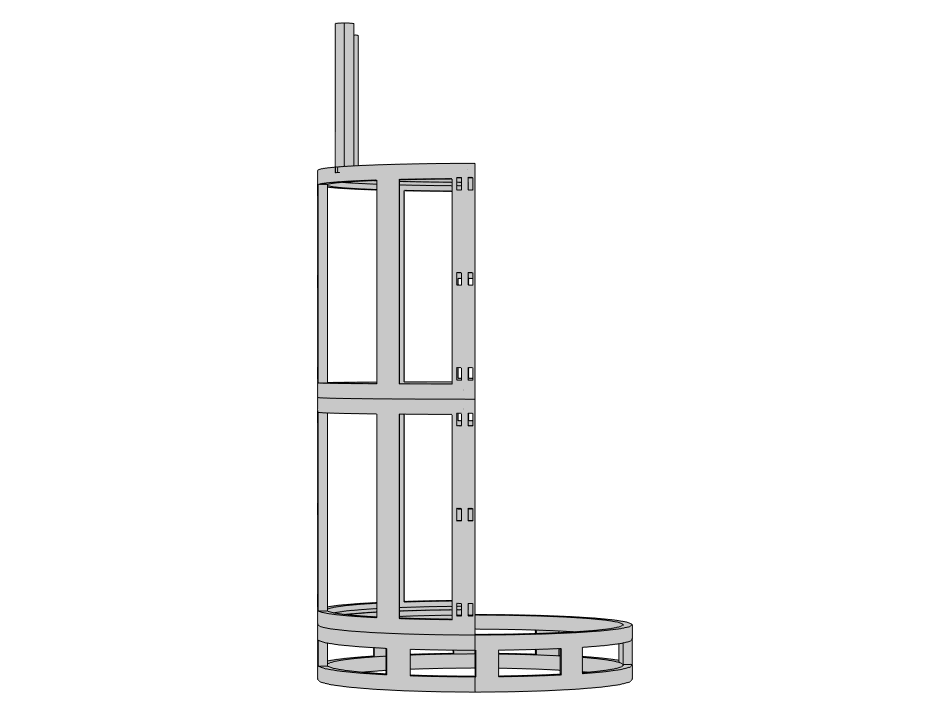

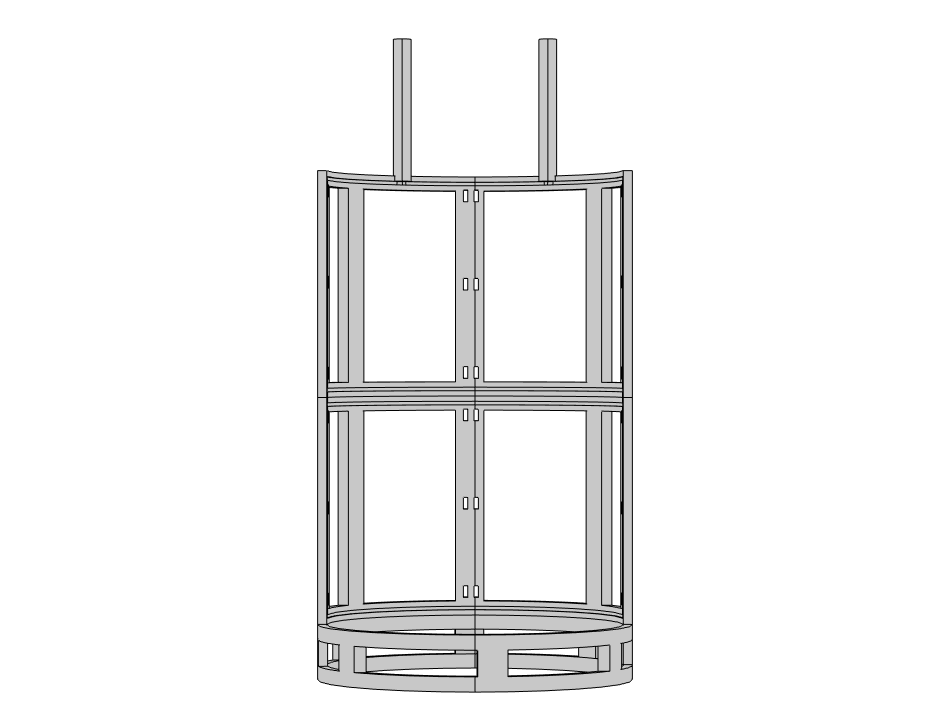

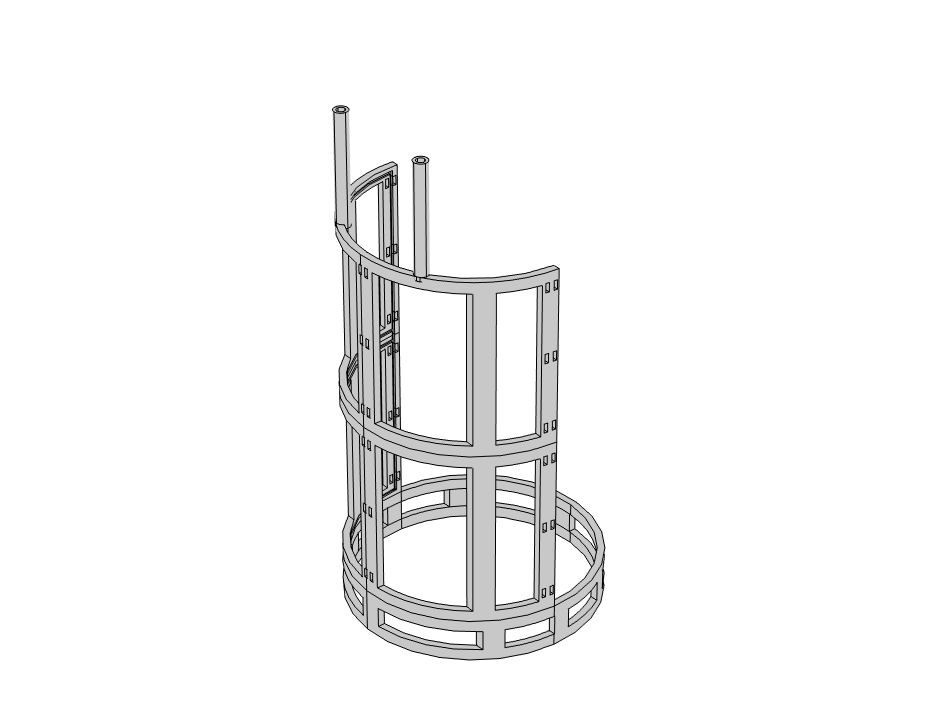


**A**

81 mm

139 mm


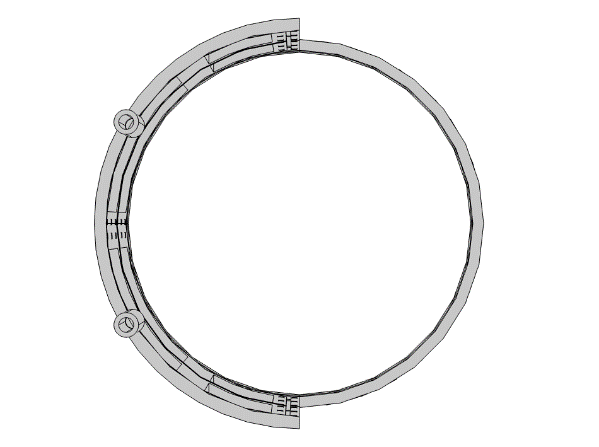
0

86 mm


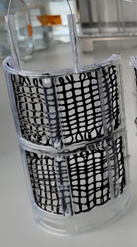

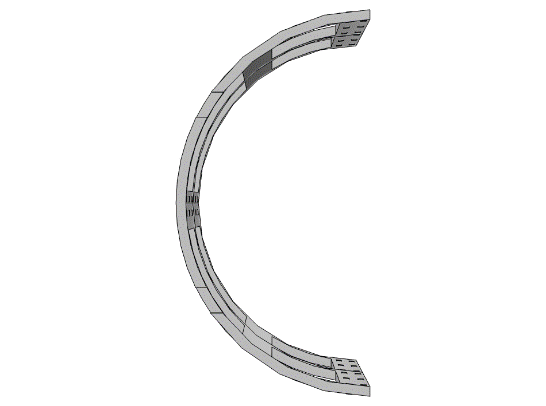

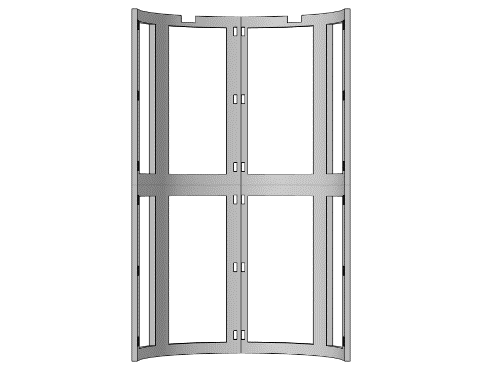

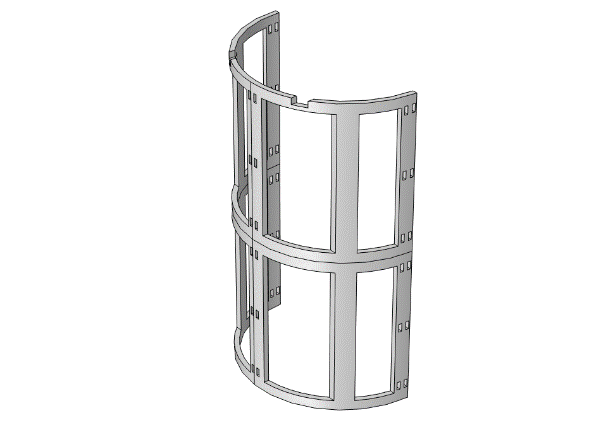

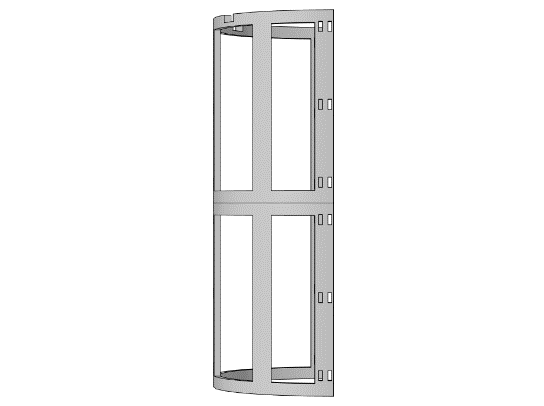


**C**

**B**

75 mm

124 mm

79 mm

**SI Figure 6:** Architecture of the 3D-printed resin holder for fixing the carbon fibre fabric cathode material in the electrobioreactors: (A) is the outer part and (B) is the inner part of the holder. (C) Carbon fiber fabric material and titanium wire acting as current collector are fixed between both parts with using cable ties.

**SI Table 1:** Resulting electrode potentials (main manuscript, Figure 3) when applying different currents in electrobioreactors for microbial electrosynthesis from CO_2_ using *C. ljungdahlii.*

| **Applied current**  **mA** | **Carbon rod cathode potential**  **V vs. Ag/AgCl** | **Carbon fibre fabric cathode potential**  **V vs. Ag/AgCl** |
| --- | --- | --- |
| -5 mA | -0.87 ± 0.04 | -0.94 ± 0.05 |
| -20 mA | -0.95 ± 0.03 | -1.02 ± 0.01 |
| -40 mA | -1.09 ± 0.09 | -1.10 ± 0.07 |
| -60 mA | -1.22 ± 0.13 | -1.12 ± 0.06 |
| -80 mA | -1.26 ± 0.14 | -1.19 ± 0.08 |

**SI Table 2:** Gradient of HPLC measurement of the amino acid compounds glycine and ethanolamine.

| **[min]** | **A [%]** | **B [%]** |
| --- | --- | --- |
| 1 | 85 | 15 |
| 40 | 65 | 35 |
| 41 | 40 | 60 |
| 46 | 40 | 60 |
| 47 | 85 | 15 |
